# Smoking Prevalence and its Association with Work-Related Factors in an Electricity and Gas Company

**DOI:** 10.1192/j.eurpsy.2024.827

**Published:** 2024-08-27

**Authors:** I. Sellami, A. Feki, A. Abbes, M. A. Ghrab, K. Jmal Hammami, M. L. Masmoudi, M. Hajjaji

**Affiliations:** ^1^Occupational medicine, Hedi Chaker Hospital; ^2^ Medecine university; ^3^Rheumatoloy, Hedi Chaker Hospital, Sfax, Tunisia

## Abstract

**Introduction:**

In the workplace, a smoke-free environment is crucial to guaranteeing the health of workers and those around them. Understanding the relationship between smoking and work is a prerequisite for implementing effective tobacco control measures.

**Objectives:**

To assess the prevalence of smoking in an electricity and gas company in Sfax and to determine the relationship between workers’ nicotine dependence and perceived workload.

**Methods:**

We conducted a cross-sectional survey evaluating the smoking behavior of 100 employees of an electricity and gas company. The survey was carried out from July to December 2022 using a two-part questionnaire. The first part was completed by the participants, and the second was administered by the interviewer. Nicotine dependence was assessed using the Fagerström test, while perceived workload was evaluated using the raw NASA-TLX questionnaire.

**Results:**

Our study population consisted of 82 male participants. Active smoking was reported by 45.1% of participants. Among smokers, 40.5% had moderate to high nicotine dependence as assessed by the Fagerström test. According to the raw NASA-TLX questionnaire, the mean scores for mental, physical, and temporal demands were 88.8±13.5, 63.6±24.7, and 59.1±28.4, respectively. The mean scores for effort, performance, and frustration were 83.8±14, 85.4±13.1, and 34.5±28.1, respectively. Bivariate analysis indicated an inverse correlation between nicotine dependence and physical demands at work. However, a significant positive correlation was found between nicotine dependence and frustration at work.

**Conclusions:**

Smoking among electricity and gas company workers is a prevalent issue, highlighting the urgent need for smoking cessation interventions. The association of smoking with perceived workload underscores the importance of preventive measures to reduce work-related stress.

**Disclosure of Interest:**

None Declared

